# In Situ Strain and Damage Monitoring of GFRP Laminates Incorporating Carbon Nanofibers under Tension

**DOI:** 10.3390/polym10070777

**Published:** 2018-07-16

**Authors:** Yanlei Wang, Yongshuai Wang, Baoguo Han, Baolin Wan, Gaochuang Cai, Ruijuan Chang

**Affiliations:** 1 State Key Laboratory of Coastal and Offshore Engineering, School of Civil Engineering, Dalian University of Technology, Dalian 116024, China; wangyanlei@dlut.edu.cn (Y.W.); wangys@mail.dlut.edu.cn (Y.W.); hanbaoguo@dlut.edu.cn (B.H.); hit1127@163.com (R.C.); 2 Department of Civil, Construction and Environmental Engineering, Marquette University, Milwaukee, WI 53201, USA; 3 Laboratory of Solid Structures, University of Luxembourg, L1359 Luxembourg, Luxembourg; gaochuang.cai@uni.lu

**Keywords:** glass fiber-reinforced polymer (GFRP), carbon nanofibers (CNFs), damage, strain, monitoring

## Abstract

In this study, conductive carbon nanofibers (CNFs) were dispersed into epoxy resin and then infused into glass fiber fabric to fabricate CNF/glass fiber-reinforced polymer (GFRP) laminates. The electrical resistance and strain of CNF/GFRP laminates were measured simultaneously during tensile loadings to investigate the in situ strain and damage monitoring capability of CNF/GFRP laminates. The damage evolution and conduction mechanisms of the laminates were also presented. The results indicated that the percolation threshold of CNFs content for CNF/GFRP laminates was 0.86 wt % based on a typical power law. The resistance response during monotonic tensile loading could be classified into three stages corresponding to different damage mechanisms, which demonstrated a good ability of in situ damage monitoring of the CNF/GFRP laminates. In addition, the capacity of in situ strain monitoring of the laminates during small strain stages was also confirmed according to the synchronous and reversible resistance responses to strain under constant cyclic tensile loading. Moreover, the analysis of the resistance responses during incremental amplitude cyclic tensile loading with the maximum strain of 1.5% suggested that in situ strain and damage monitoring of the CNF/GFRP laminates were feasible and stable.

## 1. Introduction

Fiber-reinforced polymer (FRP) composites are gaining increased importance as high performance structural materials in aerospace, automobile industries and civil infrastructure due to their high specific modulus and strength, good thermal stability and durability [1,2,3,4,5,6,7,8,9]. However, FRP composites usually exhibit gradual degradation of mechanical properties during service, which is induced by the initiation and propagation of different levels of damages, such as matrix microcracks, interfacial debonding, delamination and fiber breakage [10,11]. Predicting these damage modes in advance will improve the reliability of FRP composites and prevent them from catastrophic structural failure. Moreover, glass fiber-reinforced polymer (GFRP) composites are the most-widely used FRP among composite materials globally due to their high performance to price ratio [12,13]. Therefore, the strain and damage monitoring of GFRP composites during their long-term service is of significant interest.

Conventional strain sensors, such as active piezoelectric, fiber optical and acoustic emission sensors, have been widely used in structural health monitoring (SHM) [14,15,16]. However, the utilization of these sensors for FRP composites’ monitoring is often limited due to some drawbacks including stress concentrations around the embedded sensors, high cost, poor durability and fragility. Over the last few decades, in situ electrical resistance change (ERC) measurement has been regarded as a nondestructive method for strain and damage monitoring of FRP composites. Numerous research works have been performed to investigate the strain and damage monitoring of carbon fiber-reinforced polymer (CFRP) composites by using the ERC technique [17,18,19,20,21]. For example, Wang et al. [20] demonstrated the self-sensing of flexural strain and damage of CFRP composites by measuring the direct current electrical resistance. Therefore, monitoring the structural health of CFRP composites by means of electrical conductivity is feasible through exploiting the inherent conductivity of the carbon fiber. The main advantage of this method is that it does not require expensive equipment for instrumentation. However, this method is not available for the GFRP composites with negligible conductivity. Therefore, extending the application of ERC to the structural health monitoring of non-conductive GFRP composites is a daunting, but meaningful challenge.

Fortunately, with the rapid development of nanotechnology, the advent of carbon nanomaterials, such as carbon nanotubes (CNTs) and carbon nanofibers (CNFs), has enabled the insulated composites to be turned into electrically-conductive ones, which opens a new perspective for the development of both structural reinforcement and monitoring application at the nanoscale level. As conductive fillers for FRP composites, nanomaterials can not only improve the mechanical properties of the composites, but also endow sensing capabilities to FRP composites by providing conductive networks [22,23,24,25]. Considerable efforts have been made toward adding CNTs to GFRP by three methods: (1) dispersing CNTs into the matrix via sonication or high shear mixing [2,26,27,28,29]; (2) depositing CNTs on the glass fiber surface [12]; and (3) embedding CNT fibers into GFRP composites [30,31]. For instance, in the study of Li et al. [2], a small amount of CNT-Al_2_O_3_ hybrids was dispersed into the matrix of GFRP composites to serve as an in situ sensor to monitor the initiation and propagation of the damage under mechanical loading. Their results suggested that the resistance response could be classified into different distinguished stages to identify various failure modes of the GFRP composites. However, the strain and damage monitoring of the composites under cyclic loading were not explored in their research. Gao et al. [8] investigated the monitoring application of GFRP composites by depositing CNTs on a glass fiber surface. The results confirmed that the techniques of ERC measurement incorporating CNTs could be applicable to in situ strain and damage sensing of GFRP composites. Although the conductive fillers on fiber surface are very sensitive to the fracture of the loading-carrying fibers, they provide less information about the cracking development in the matrix, where the microscale damages are initiated and developed. In addition, CNFs have become a potential alternative to CNTs in the health monitoring of FRP composites, especially in large-scale applications due to their relatively simple fabrication, easy dispensability and low cost compared to CNTs [32,33]. Therefore, it is worth studying the feasibility and repeatability of in situ strain and damage monitoring of GFRP laminates fabricated with CNFs/epoxy composites under monotonic and cyclic loadings.

In this study, conductive GFRP laminates were prepared by using a CNF/epoxy mixture as the matrix for the composites and the wet lay-up technique. The electrical properties of the CNF/GFRP laminates were investigated first, followed by the discussion of the conductive mechanisms based on the tunneling conduction and percolation conduction theories. Then, the electrical resistances of the CNF/GFRP laminates were tracked during monotonic, constant cyclic and incremental cyclic loadings to explore their in situ strain and damage monitoring capabilities.

## 2. Materials and Methods

### 2.1. Materials and Specimens

The unidirectional glass fiber fabric used in this study was the HITEX-G430E glass fibers with an areal density of 430 g/m^2^ produced by Nanjing Haituo Composite Materials Co., Ltd., Nanjing, China. The warp glass yarn (an areal density of 400 g/m^2^) was fixed in the fabric with the weft polyester yarn (an areal density of 30 g/m^2^). The nominal thickness of the glass fiber fabric is 0.169 mm. The diameter of the fiber is about 16 μm. Epoxy resin manufactured by Tianjin Swancor Wind Power Materials Co., Ltd., Tianjin, China, was used as the matrix of the GFRP composites. It was mixed with SWANCOR 2511-1A (main agent) and SWANCOR 2511-1BS (curing agent) with a ratio of 10:3 by weight. This epoxy resin has low viscosity, moderate gel time, nice mechanical properties, a high heat deflection temperature (HDT) and good wettability to glass fibers. The CNFs (Pyrograf-III PR-24-XT-HHT) with an average diameter of 100 nm and a length of 50–200 μm were supplied by Pyrograf Products, Inc., Cedarville, OH, USA. They were fully graphitized at 3000 °C and contain a low content of catalyst. The CNFs in the amounts of 0.5%, 1.0%, 1.5%, 2.0% and 3.0% by weight of epoxy resin (i.e., 0.29%, 0.58%, 0.87%, 1.16% and 1.74% by volume of epoxy resin, respectively) were applied into epoxy resin, respectively. The corresponding CNF/GFRP laminates are called L_0.5_, L_1.0_, L_1.5_, L_2.0_ and L_3.0_, respectively. Acetone was used as the diluting agent in the amount of 2% by volume of the composites. A copper sheet with a thickness of 0.03 mm and a width of 5 mm was used as the electrode. Conductive copper paint was used to ensure good contact between the specimens and the electrodes.

**Figure 1:**
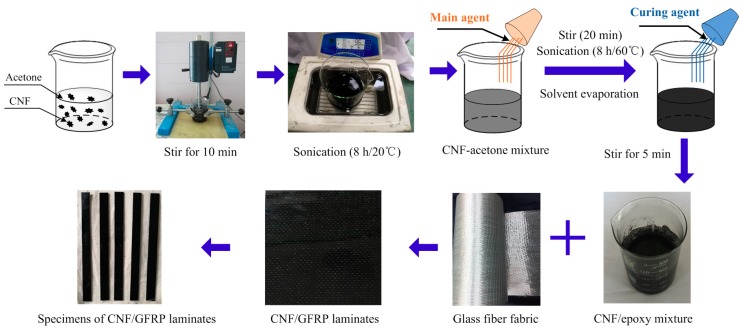
Preparation processes of CNF/GFRP laminates.

**Figure 2:**
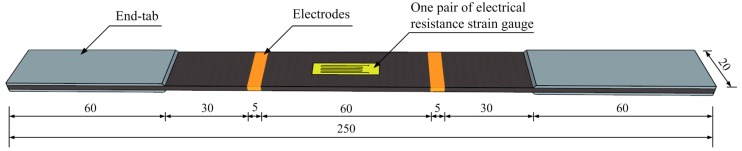
Schematic of CNF/GFRP laminates (units in mm).

A high degree of CNF dispersion in the epoxy matrix is crucial for the formation of electrically-conductive networks that penetrate throughout GFRP composites. The general production processes of the CNF/GFRP laminates are as follows (illustrated in Figure 1). (1) The CNFs were firstly dried in an oven at 60 °C for 30 min to remove the moisture. Five amounts of CNFs (i.e., 0.5, 1.0, 1.5, 2.0 and 3.0 wt %) were separately dispersed in the acetone solution by using a mechanical stirrer at a high speed (1500 r/min) for 10 min and then sonicated by a ultrasonic cleaner for 8 h at 20° C to get the CNF-acetone mixture. (2) Heated (at 60 °C for 5 min) SWANCOR 2511-1A (main agent) was dissolved in the CNF-acetone mixture by using the mechanical stirrer at a high speed (1500 r/min) for 20 min and ultrasonically dispersing at 60 °C for 8 h to get a CNF-acetone-epoxy (main agent) mixture. (3) The CNF-acetone-epoxy (main agent) mixture was placed in a vacuum oven to remove the air bubbles and the acetone. (4) SWANCOR 2511-1BS (curing agent) was added in the mixture obtained in Step 3 via stirring at a low speed (500 r/min) for 5 min to get the CNF/epoxy mixture. The purpose of the low speed is to avoid introducing a large amount of air bubbles into the mixture. (5) The CNF/GFRP laminates were prepared by wet lay-up. Glass fiber fabrics were properly stacked into four plies along the fiber direction to ensure significant Poisson effects in both the width and thickness direction. The CNF/epoxy mixture was poured onto a layer of glass fiber fabric and spread uniformly with a roller. After the fabric was completely saturated by the CNF/epoxy mixture, the remaining three layers of the fabric were infiltrated by the repeated operations. Finally, each layer of the fabrics was uniformly impregnated with the CNF/epoxy mixture. (6) CNF/GFRP laminates were pre-cured at room temperature for 24 h followed by a post-cure for an additional 8 h at 60 °C. (7) Test specimens were cut from the cured CNF/GFRP laminates. Two 5 mm-wide copper sheets were glued at the surface of each CNF/GFRP specimen using a conductive copper paint. Figure 2 shows the dimensions of the specimens and the layout of the electrodes. The thickness of the CNF/GFRP laminates is 1.686 mm. The volume fraction of the laminates is about 40.1%.

### 2.2. Measurement

#### 2.2.1. Electrical Measurement

CNF/GFRP laminates with CNFs contents of 0, 0.5, 1.0, 1.5, 2.0 and 3.0 wt % were used for testing the electrical properties. Three samples of each type of CNF/GFRP laminate were prepared. The direct current (DC) electrical resistance of CNF/GFRP laminates was measured by 2-electrode method using a digital multimeter. The constant voltage provided by the digital multimeter is 10 V. The main reason for using 2 electrodes instead of 4 electrodes is that 2 electrode are more versatile in terms of implementation than that 4 electrodes [34]. The two copper sheets were used as the electrodes. The electrical resistivity (*ρ*), i.e., the resistance per unit volume in the bulk material, is determined by:

(1) $$\rho =R\frac{A}{L}$$

where *R* is the measured electrical resistance, *A* is the cross-sectional area of the specimen and *L* is the distance between the two copper electrodes.

#### 2.2.2. In Situ Monitoring Measurement

**Figure 3:**
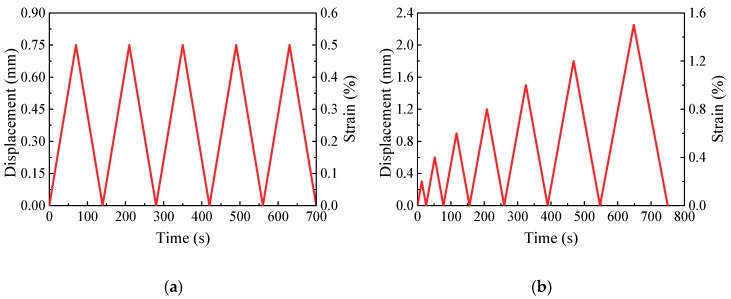
Cyclic loading paths: (**a**) Loading Type I; (**b**) Loading Type II.

**Figure 4:**
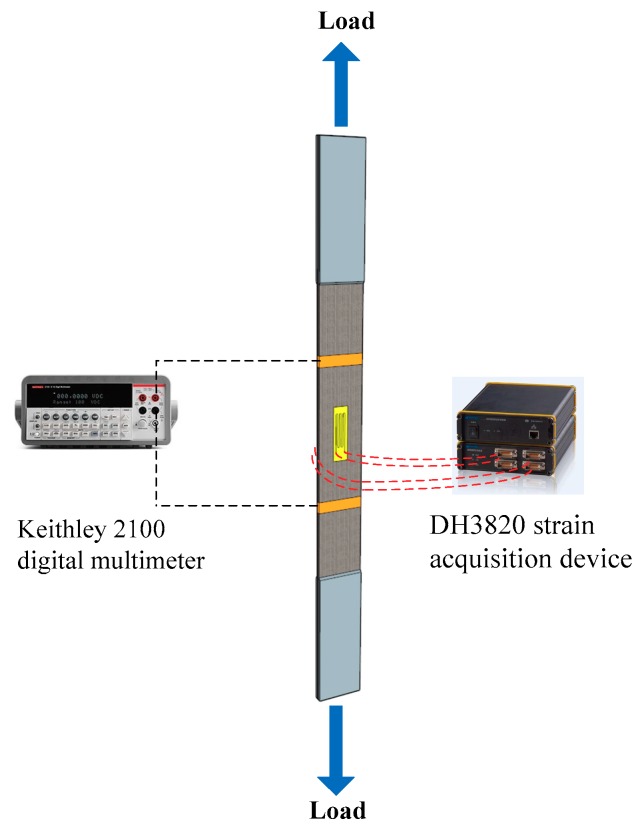
Setup of the in situ monitoring test.

According to the results of the electrical tests, CNF/GFRP laminates containing 1.0 and 1.5 wt % of CNFs, which were close to the percolation threshold of CNFs content (0.86 wt %), were selected for in situ monitoring tests. The in situ monitoring experiments were performed by applying monotonic and cyclic uniaxial tensile loadings and simultaneously measuring strains and electrical resistances of the CNF/GFRP specimens. The loads were applied by a servo-hydraulic testing machine (Model WDE-200E, Jinan Gold Testing Machines Inc., Jinan, China) under displacement control. The electrical resistance measurement was carried out to monitor the damage evolution of CNF/GFRP laminates in situ during monotonic tensile loading. Two loading paths were applied for the cyclic loading. Loading Type I (as shown in Figure 3a) consisted of five loading-unloading cycles with constant strain amplitudes of 0.5%. Loading Type II (as shown in Figure 3b) consisted of seven cycles with increasing strain amplitudes of 0.2%, 0.4%, 0.6%, 0.8%, 1.0%, 1.2% and 1.5%. During the test process, the DC resistances were tested via the Keithley 2100 (Keithley Instruments, Inc., Cleveland, OH, USA) digital multimeter, and the strains were monitored by one pair of electrical resistance strain gauges, which were bonded at the two sides of the middle section of the specimens (as shown in Figure 2). The DH3820 strain acquisition device (Donghua Testing Technology Co., Ltd., Zhenjiang, China) was employed to record strain data. The test setup of the in situ monitoring test is shown in Figure 4. In this study, six specimens of L_1.0_ and L_1.5_ CNF/GFRP laminates were used for the in situ monitoring test. For each type of laminate, three specimens were tested under monotonic tensile loads until their failures, and the remaining three specimens were first subjected to constant amplitude cyclic tensile loading and then subjected to incremental amplitude cyclic tensile loading to simulate realistic loading conditions. The responses of the specimens subjected to cyclic tensile loadings were also used to verify the stability of the in situ monitoring properties. Moreover, sand paper was applied between the clamps and the specimens to not only improve the gripping, but also provide electrical insulation between the machine frame and the samples.

Because the deformation of the specimens under tension is small, the changes of the deformation in the separation (*L*) between the two electrodes can be neglected [35]. Therefore, the fractional change in electrical resistivity (FCR), *f*, is equivalent to the fractional change in electrical resistance:

(2) $$f=\frac{\Delta \rho }{\rho_{0}}=\frac{\Delta R\times A/L}{R_{0}\times A/L}=\frac{\Delta R}{R_{0}}=\frac{R_{t}-R_{0}}{R_{0}}$$

where ∆*ρ* and ∆*R* are the changes in electrical resistivity and resistance, respectively; *ρ*_0_ and *R*_0_ are the initial resistivity and resistance, respectively, of the specimens without loading; *A* is the cross-sectional area of the specimens; *L* is the separation between the two electrodes; and *R_t_* is the resistance at time *t* during the test.

#### 2.2.3. Microstructure Characterization

The microstructure and morphology of the fabricated CNF/GFRP laminates were observed by a scanning electron microscope (SEM) (Nova NANOSEM450, FEI Inc., New York, NY, USA) and optical microscopy to investigate the failure mechanisms of the specimens. In situ microstructure observations during the tensile testing are not possible. Therefore, the CNF/GFRP laminates specimens were carefully removed from the tensile machine after unloading, when the specimens were loaded to the pre-set strain levels. The samples for microscopic observation by SEM were cut from the loaded CNF/GFRP laminate specimens. For each type of observation, five samples were investigated by SEM.

## 3. Results and Discussions

### 3.1. Electrical Properties of CNF/GFRP Laminates

**Figure 5:**
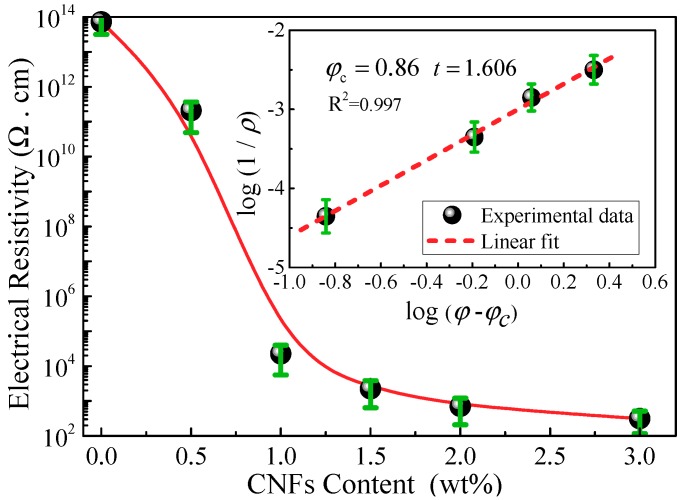
Electrical resistivity of CNF/GFRP laminates as a function of CNF content (inset: logarithm of conductivity as a function of the logarithm of the reduced weight fraction and fit to a line).

Figure 5 shows the electrical resistivity of CNF/GFRP laminates as a function of CNF content. It is observed that a significant decrease of 10 orders of magnitude of the resistivity happened when the content of CNFs increased from 0–1.0 wt %, and then, the resistivity decreased at a much smaller rate at higher contents of CNFs. The sharp change of resistivity indicates the formation of a percolating network in the CNF/GFRP composites. In order to confirm the percolation threshold of CNF content in CNF/GFRP laminates from theoretical investigations, the classical power law was investigated [2].

(3) $$\rho =B{\left(\varphi -\varphi_{c}\right)}^{-t}\;\mathrm{for}\;\varphi \geq \varphi_{c}$$

where *B* is a constant; *φ* is the weight fraction of CNF content; *φ_c_* is the critical weight fraction of CNF content, i.e., the percolation threshold (when *φ* is larger than *φ_c_*, CNFs can form conductive paths in the nanocomposite); and *t* is the critical exponent related to the dimensionality of the system. The best fit of the experimental resistivity data to log-log plots of the percolation theory (the inset in Figure 5) gives the results shown in Table 1, i.e., the percolation threshold, *φ_c_*, was 0.86 wt % and the critical exponent, *t*, was 1.606, which is close to that of a three-dimensional random system (*t* = 1.94) [36].

**Figure 6:**
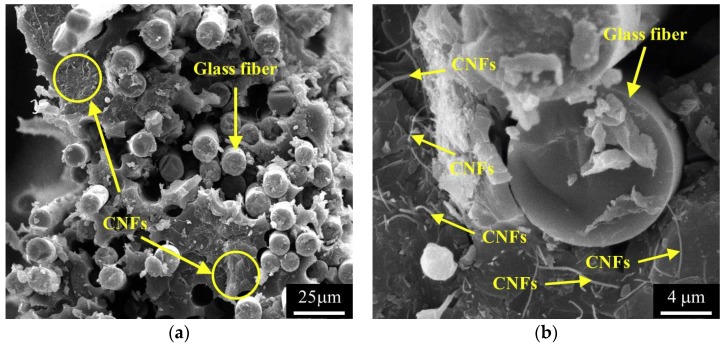
SEM images of fracture surfaces: (**a**) L_1.0_; (**b**) L_1.5_.

The electrical properties of the CNF/GFRP laminates agree with the paths theory, i.e., the number of conduction paths between the CNFs increases with the increasing of CNF content. Figure 6 shows the typical microstructures of the CNF/GFRP laminates. As shown in Figure 6, glass fibers are surrounded by CNF/epoxy composites with a homogeneous hybrid structure. Most CNFs can be uniformly dispersed in the epoxy matrix and penetrated the glass fiber bundles to form a conductive network throughout the hybrid structure. When the CNF contents of CNF/GFRP laminates were relative low (below 0.86 wt % in this paper), the conductive paths were sparse, so the tunneling conduction between the adjacent CNFs is the major cause for the decrease of the resistivity. When the contents of CNFs in CNF/GFRP laminates were relative high (higher than 0.86 wt %), much more conductive networks were formed. Therefore, the electrical conductivity of the CNF/GFRP laminates increases dramatically through the percolation conduction, which is the major cause for the decrease of the electrical resistivity of the CNF/GFRP laminates.

### 3.2. In Situ Monitoring of CNF/GFRP Laminates

#### 3.2.1. In Situ Damage Monitoring under Monotonic Tensile Loading

**Figure 7:**
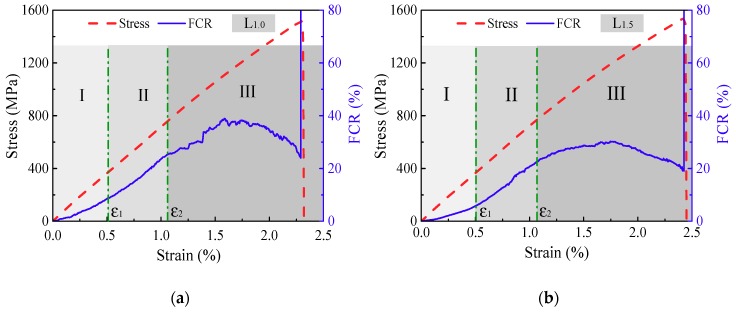
In situ damage monitoring of (**a**) L_1.0_ and (**b**) L_1.5_ under monotonic tensile loading. FCR, fractional change in electrical resistivity.

Figure 7 shows the typical FCR and strain responses of L_1.0_ and L_1.5_ CNF/GFRP laminates under monotonic tensile loading until their ultimate failure. It can be seen from Figure 7 that the stress vs. stain curves of L_1.0_ and L_1.5_ exhibit a nearly linear relationship, while the FCR vs. strain curves vary with a discontinuous slope. The reason is that the load of the specimen is mainly carried by the glass fibers, whereas the electrically-conductive networks formed by the internal CNFs/epoxy composites are successively destroyed due to the damage during monotonic tensile loading. The FCR responses can be classified into three stages according to the two critical strains (*ε*_1_ and *ε*_2_ shown in Figure 7) corresponding to the different damage mechanisms. The critical strains can be determined by identifying the changes of the slopes of the FCR-strain curves.

**Figure 8:**
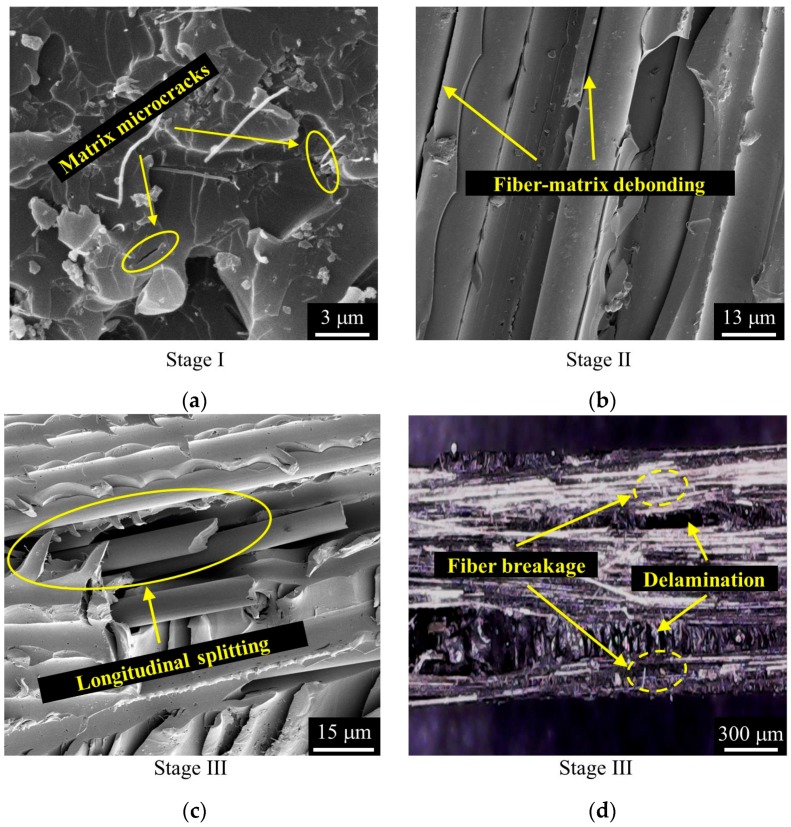
SEM and optical morphologies of damage initiation and development in CNF/GFRP laminates: (**a**) matrix microcracks; (**b**) fiber-matrix interfacial debonding; (**c**) longitudinal splitting; (**d**) delamination and fiber breakage.

**Figure 9:**
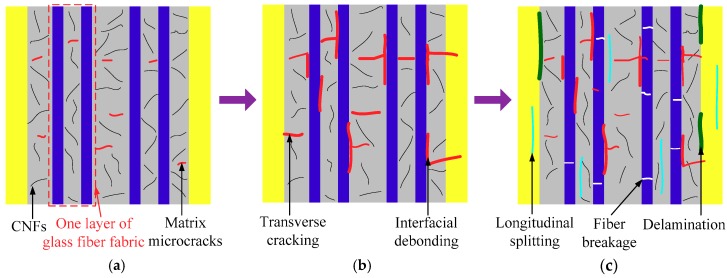
Illustration of the damage evolution of CNF/GFRP laminates during monotonic loading: (**a**) initiation of matrix microcracks; (**b**) transverse cracking and fiber-matrix interfacial debonding; (**c**) longitudinal splitting, delamination and glass fiber breakage.

The macroscopic damages of FRP composites generally include matrix cracking, interfacial debonding, transverse cracking, longitudinal splitting, delamination, as well as local fiber breakage and pull-out [27]. Considering the damage mechanisms, the FCR responses of the CNF/GFRP laminates in three stages shown in Figure 7 can be explained. The representative SEM and optical morphologies of damage initiation and development in CNF/GFRP laminates are shown in Figure 8. The schematics in Figure 9 present the general damage evolution of CNF/GFRP laminates. It should be noted that two columns of fibers are used to represent one layer of glass fiber fabric in the schematic. In Stage I, the FCR response to strain is almost linear, corresponding to only a few matrix-dominated microcracks (as shown in Figure 8a). Microcrack closing may be possible for unloading specimens. During Stage I, the microcracks that could be observed mainly came from the pre-existing defects in the sample. With the continuous increase of the strain, when *ε*_1_ *< ε* < *ε*_2_ (in Stage II), the slope of the FCR-strain curve began to increase. In this stage, the development of some microcracks could lead to transverse cracking and interfacial debonding (as shown in Figure 8b). The fiber-matrix interfacial debonding and transverse cracking may cause more destruction of the conductive networks in CNF/GFRP laminates. This might be the main reason for the rapid increase of FCR values in Stage II shown in Figure 7. During Stage III (*ε* > *ε*_2_), it is interesting to observe that the FCR starts to increase slowly compared to Stage II and then decreases with the gradual increase of the strain. The final sharp increase of FCR indicates the final failure of the laminates. In this stage, besides the transverse cracking and interfacial debonding, the longitudinal splitting (as shown in Figure 8c), delamination and glass fiber breakage (as shown in Figure 8d) would take place, which could further destroy the conductive networks in the laminates. However, the decrease of resistivity of the CNF/GFRP laminate in Stage III may be attributed to the reformation of the conductive networks. When the strain entered Stage III, Poisson’s contraction of the matrix became more obvious. Considering the FCR variation in Stage III shown in Figure 7, it is considered that Poisson’s contraction of the matrix could have played a significant role in the closing of microcracks [37]. This may cause the CNFs to be close enough to endow the tunneling effect, thereby reforming the conductive networks. In addition, the reorientation of CNFs is also helpful for the reformation of the conductive networks [38]. Therefore, there were two competitive factors mainly influencing the electrical resistivity in Stage III. Damage development caused the destruction of conductive networks in the laminates, leading to the increase of the electrical resistivity. Poisson’s contraction of the matrix and the reorientation of CNFs facilitated the reformation of the conductive networks, resulting in a decrease of the electrical resistivity. However, the latter had a much greater influence on the resistivity change than the former, which could be used to explain the slight increase and then decrease of the FCR values during Stage III. Overall, the FCR responses observed in Stages II and III can be used as early warning of the damage of CNF/GFRP composites.

#### 3.2.2. In Situ Strain Monitoring under Constant Amplitude Cyclic Tensile Loading

**Figure 10:**
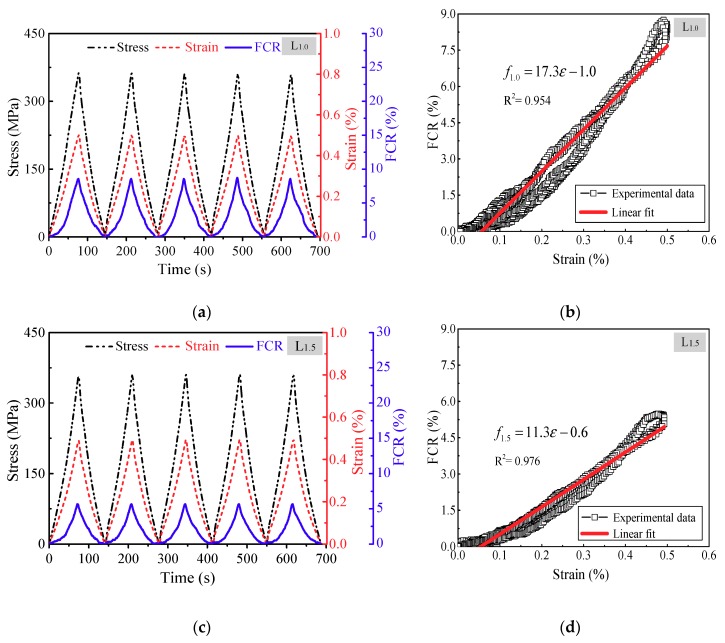
Strain monitoring of CNF/GFRP laminates under constant amplitude cyclic tensile loading: (**a**) FCR, strain and stress vs. time for L_1.0_; (**b**) FCR vs. strain for L_1.0_; (**c**) FCR, strain and stress vs. time for L_1.5_; (**d**) FCR vs. strain for L_1.5_.

The descriptions in the previous section have demonstrated that CNF/GFRP laminates have excellent in situ damage monitoring abilities under monotonic tensile loading. In order to evaluate the feasibility and repeatability of in situ strain monitoring of CNF/GFRP laminates, constant amplitude cyclic tensile loading was applied to L_1.0_ and L_1.5_. Due to the instability of the conductive networks inside the CNF/GFRP laminates in the initial state, the FCR-strain curve of the cyclic loading test would show significant drift during the initial few cycles. Therefore, pre-tension treatment, i.e., five repeated cycles, for each specimen was carried out by applying cyclic preconditioning strain before the cyclic tensile loading test in order to achieve a stable conductive state. Figure 10 shows in situ strain monitoring of L_1.0_ and L_1.5_. The maximum strain of cyclic tensile loading was 0.5%, which was in Stage I, as discussed in the previous section. Therefore, there should not have been obvious damage initiated in the laminates. It can be observed from Figure 10a,c that the instantaneous response of FCR closely followed the change of strain/stress, which indicates that the FCR values of CNF/GFRP laminates varied synchronously with the applied strain/stress. In the continuous stretching-releasing cycles, both L_1.0_ and L_1.5_ essentially recovered their resistance after releasing, suggesting good repeatability of the strain monitoring property of the CNF/GFRP laminates during low deformation.

**Figure 11:**
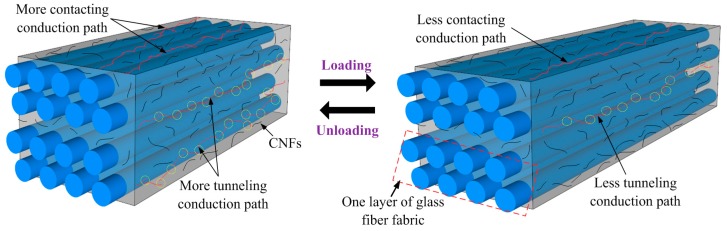
Schematic diagram of the piezoresistive mechanisms of CNF/GFRP laminates.

The in situ strain monitoring capability of the CNF/GFRP laminates is based on the principle of the piezoresistive properties of the nanocomposites. Figure 11 shows the schematic diagram of the piezoresistive mechanisms of CNF/GFRP laminates. It should be noted that two rows of fibers were used to represent a layer of fiber fabric in the schematic. Since the CNFs contents of L_1.0_ and L_1.5_ were larger than the percolation threshold (0.86 wt %), abundant conductive networks could be established in these CNF/GFRP laminates. The piezoresistive mechanisms were related to tunneling conduction and contacting conduction (also named percolation conduction). When the CNF/GFRP was subjected to tensile loading, the epoxy matrix experienced a large deformation in the direction of tensile loading, leading to an increase of the distance between the adjacent CNFs. In this circumstance, less conductive paths created by tunneling conduction and contacting conduction among the CNFs would be formed, as shown in Figure 11, which would result in an increase of the resistivities of CNF/GFRP laminates. During unloading, the situation was the opposite. Therefore, during the constant amplitude loading/unloading, the leaving/approaching of the adjacent CNFs caused the increase/decrease of the resistivity of the laminates. Overall, the FCR varied synchronously and reversibly with the applied strain, which confirms the outstanding in situ strain monitoring capability of CNF/GFRP laminates.

As shown in Figure 10b,d, there is a positive correlation between the FCR and strain with a low noise level under the five-cycle tensile loading. For a more complete understanding of in situ strain monitoring of CNF/GFRP laminates, the relevant parameters including gauge factor, linearity, repeatability and hysteresis were investigated. The relationships between input value *ε* (strain value) and output value *f* (FCR value) of the laminates were obtained using the linear fit by least squares method (shown in Figure 10b,d), which can be expressed as:

(4) $$f_{1.0}=17.3\varepsilon -1.0$$

(5) $$f_{1.5}=11.3\varepsilon -0.6$$

Gauge factor, *S*, also called the strain sensitivity coefficient, which is defined as the ratio between the fractional change of electrical resistance (Δ*R/*$R_{0}$) and the strain (*ε*), is given by Equation (6).

(6) $$S=\frac{\Delta R/R_{0}}{\varepsilon }$$

Linearity, *E*, is the offset between strain-FCR curves and the fitted regression line, which is defined as:

(7) $$E=\frac{\Delta_{\max}}{f_{F.S}}\times 100\%$$

where Δ_max_ is the maximum deviation of strain-FCR curves from the fitted regression line and *f_F,S_* is the output range. Linearity is also known as nonlinearity error, and a smaller value indicates a better linear feature.

Repeatability, *R*, is the degree of the repetition of the output values under the same conditions, which is expressed as:

(8) $$R=\frac{\Delta R_{\max}}{f_{F.S}}\times 100\%$$

where Δ*R*_max_ is the maximum repeat difference, which is the difference of the FCR for the same strain in the same process during loading and unloading.

Hysteresis, *H*, means that the resistivity of the laminates does not completely recover its initial value after unloading in certain cases, which is determined by:

(9) $$H=\frac{\Delta f_{\max}}{f_{F.S}}\times 100\%$$

where Δ*f*_max_ is the maximum difference of FCR in all processes during the cyclic loading.

The results of the above parameters are shown in Table 2. It can be observed from Table 2 that although L_1.5_ has a lower sensitivity, it possesses lower linearity, repeatability and hysteresis compared to L_1.0_. The plot in Figure 10d also demonstrates good linearity and repeatability of the piezoresistive performances of L_1.5_ by the linear relationship between the FCR and strain with relatively small scatter.

#### 3.2.3. In Situ Monitoring under Incremental Amplitude Cyclic Tensile Loading

**Figure 12:**
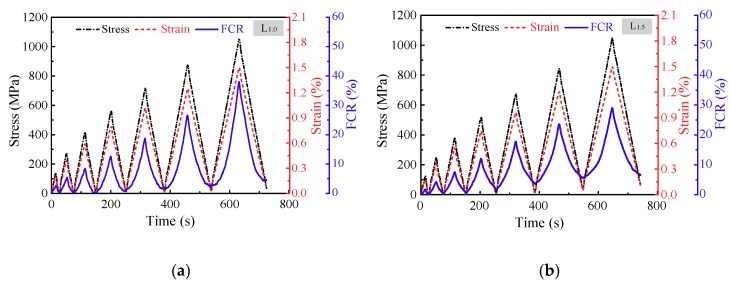
Typical in situ monitoring of (**a**) L_1.0_ and (**b**) L_1.5_ under incremental amplitude cyclic tensile loading.

**Figure 13:**
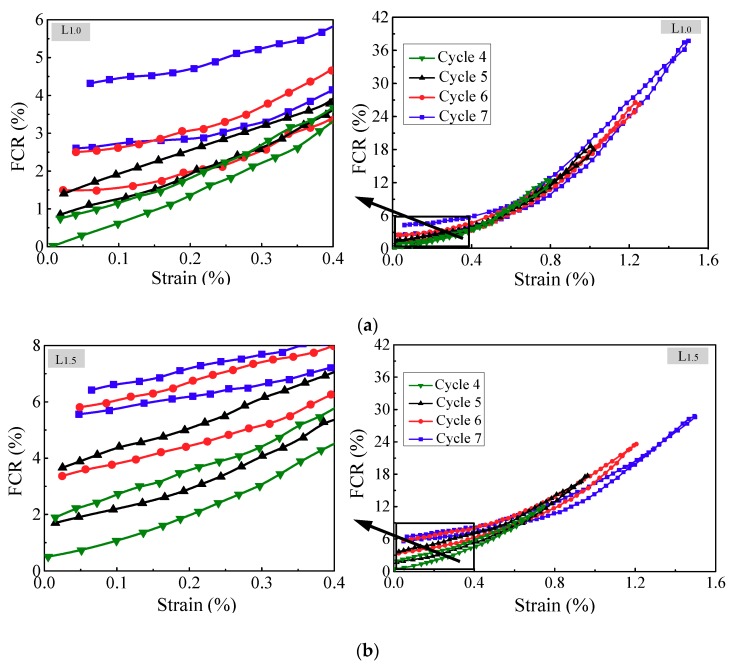
FCR vs. strain curves of (**a**) L_1.0_ and (**b**) L_1.5_ from the fourth to seventh cycle during incremental amplitude cyclic tensile loading.

In order to simulate the real loading conditions and verify the reproducibility of the strain and damage monitoring properties, incremental amplitude cyclic tensile loading was applied to CNF/GFRP laminates. The typical in situ monitoring of L_1.0_ and L_1.5_ is shown in Figure 12. The maximum strain was 1.5%, which was close to the strain corresponding to the turning point of the FCR response under monotonic loading as shown in Figure 7. It can be observed from Figure 12 that the FCR varied distinctly in response to the applied strain/stress. The FCR values increased with the increasing of loading amplitude and the decreasing of CNF content. Figure 12 reveals that the laminates could recover their resistance after the unloading from the first to the third cycle, indicating the excellent in situ strain monitoring capability of CNF/GFRP laminates during small strain (less than 0.5%). However, the residual resistance of the laminates began to appear after the unloading of the fourth cycle, suggesting the damage being initiated in the laminates. FCR vs. strain curves of L_1.0_ and L_1.5_ from the fourth cycle to seventh cycle under incremental amplitude cyclic tensile loading are illustrated in Figure 13. It is clear that there was a permanent resistance change under the unloading state in each subsequent cycle after damage was initiated. The formation and development of damage in the CNF/GFRP laminates resulted in the breakup of the conductive networks and permanent changes in electrical resistance. Therefore, CNF/GFRP laminates can be used for in situ strain and damage monitoring under incremental amplitude cyclic tensile loading.

GFRP composites have been widely used in the field of structural reinforcement. According to the research in this study, GFRP composites incorporating CNFs can in situ monitor their strain and damage. Therefore, using CNF/GFRP composites to reinforce concrete structures can not only effectively improve the bearing capacity of concrete structures, but also monitor the structural strain and damage during their long-term service. These are also subjects that our research group will follow.

## 4. Conclusions

Different contents of CNFs were dispersed in epoxy resin and then infused into four-ply unidirectional glass fiber fabric to fabricate CNF/GFRP laminates. The electrical resistance was measured during monotonic, constant amplitude cyclic and incremental amplitude cyclic tensile loadings to investigate in situ damage and strain monitoring of CNF/GFRP laminates. The damage evolution and conduction mechanisms of the CNF/GFRP laminates were also explored. The following conclusions can be drawn from this study: The percolation threshold of CNF content in the CNF/GFRP laminates was 0.86 wt % according to the classical power law. A good capability of in situ damage monitoring of CNF/GFRP laminates was proven under monotonic tensile loading till final failure. FCR responses of CNF/GFRP laminates can be classified into three stages corresponding to different damage mechanisms, i.e., matrix microcracking, transverse cracking, debonding, longitudinal splitting, delamination and fiber breakage. Piezoresistive properties of CNF/GFRP laminates, which are based on tunneling conduction and percolation conduction theories, caused the synchronous FCR responses to strain during constant cyclic tensile loading. This phenomenon confirmed the stable ability of in situ strain monitoring of the CNF/GFRP laminates. Moreover, the laminate with 1.5 wt % of CNFs exhibited better strain monitoring properties compared to that of the laminate with 1.0 wt % of CNFs. The analysis of the resistance responses during the incremental amplitude cyclic tensile loading with the maximum strain of 1.5% proves that in situ strain and damage monitoring of the CNF/GFRP laminates are feasible and stable.

## Figures and Tables

**Table 1:** The results calculated with classical percolation theory.

Constant *B*	Critical Weight Fraction *φ_c_* (wt %)	Critical Exponent *t*
7.69	0.86	1.606

**Table 2:** Parameters of in situ strain monitoring of CNF/GFRP laminates.

Parameters	Input Range (%)	Output Range (%)	Gauge Factor	Linearity (%)	Repeatability (%)	Hysteresis (%)
L_1.0_	0–0.5	0–8.7	17.4	14.2	7.9	18.5
L_1.5_	0–0.5	0–5.5	11.0	11.7	5.1	6.3
